# Single-Cell RNA-Seq and Bulk RNA-Seq Reveal Intratumoral Heterogeneity and Tumor Microenvironment Characteristics in Diffuse Large B-Cell Lymphoma

**DOI:** 10.3389/fgene.2022.881345

**Published:** 2022-05-04

**Authors:** Yang Zhao, Hui Xu, Mingzhi Zhang, Ling Li

**Affiliations:** Department of Oncology, First Affiliated Hospital of Zhengzhou University, Zhengzhou, China

**Keywords:** diffuse large B-cell lymphoma, single-cell RNA sequencing, tumor microenvironment, tumor heterogeneity, prognosis

## Abstract

**Background:** Diffuse large B-cell lymphoma (DLBCL) is the most common histologic subtype of non-Hodgkin’s lymphoma (NHL) with highly heterogeneous genetic and phenotypic features. Therefore, a comprehensive understanding of cellular diversity and intratumoral heterogeneity is essential to elucidate the mechanisms driving DLBCL progression and to develop new therapeutic approaches.

**Methods:** We analyzed single-cell transcriptomic data from 2 reactive lymph node tissue samples and 2 DLBCL lymph node biopsy tissue samples to explore the transcriptomic landscape of DLBCL. In addition, we constructed a prognostic model based on the genes obtained from differential analysis.

**Results:** Based on gene expression profiles at the single cell level, we identified and characterized different subpopulations of malignant and immune cells. Malignant cells exhibited a high degree of inter-tumor heterogeneity. Tumor-infiltrating regulatory CD4^+^ T cells showed highly immunosuppressive properties and exhausted cytotoxic CD8^+^ T cells were highly expressed with markers of exhaustion. Cell communication analysis identified complex interactions between malignant cells and other cell subpopulations. In addition, the prognostic model we constructed allows for monitoring the prognosis of DLBCL patients.

**Conclusion:** This study provides an in-depth dissection of the transcriptional features of malignant B cells and tumor microenvironment (TME) in DLBCL and provides new insights into the tumor heterogeneity of DLBCL.

## Introduction

Diffuse large B-cell lymphoma (DLBCL) is the most common histologic subtype of non-Hodgkin’s lymphoma (NHL) with highly heterogeneous genetic and phenotypic features. Gene expression profiling divides DLBCL into two distinct molecular subtypes, the activated B-cell-like and the germinal center B-cell-like subtypes (Scott et al., 2014; Reddy et al., 2017). Although the standard first-line treatment regimen (R-CHOP) results in complete and durable remission in approximately 60% of cases, relapse occurs in 30–40% of patients and refractory disease in another 10% (Friedberg 2006; Friedberg 2011). Autologous stem cell transplantation (ASCT) after salvage chemotherapy is the standard second-line treatment for relapsed or refractory (R/R) DLBCL (Gisselbrecht et al., 2010). However, half of the patients are not eligible for transplantation due to ineffective salvage therapy, and the other half relapse after ASCT (Crump et al., 2017). The prognosis of this group of patients is extremely poor and the choice of treatment options is challenging.

The journal Science selected tumor immunotherapy as the most important scientific breakthrough of 2013 (Couzin-Frankel 2013). In 2017, the U.S. Food and Drug Administration approved two chimeric antigen receptor T-cells targeting CD19 for the treatment of R/R B-cell malignancies (Dwivedi et al., 2019). Tumor immunotherapy has become a more important treatment after the development of drug resistance in DLBCL patients. Studies have shown that the tumor immune microenvironment has a great impact on the efficacy of immunotherapy (Li et al., 2018). Thus, it has become a primary task to improve the current status of DLBCL treatment with important clinical significance to deeply explore the state of tumor microenvironment (TME) and drug resistance mechanism in DLBCL patients and find new therapeutic targets for DLBCL.

Tumor cells exist in a complex microenvironment composed of infiltrating immune cells and stromal cells. These immune cells and stromal cells, together with the cytokines and chemokines they secrete, as well as the intercellular stroma and microvasculature in the nearby area, constitute a complex network of TME (Hui and Chen 2015; Shen and Kang 2018). Tumor cells maintain their survival and proliferation by communicating with the TME network, which also allows tumor cells to develop immunosuppressive mechanisms to evade immune surveillance and promote disease progression (Coupland 2011; Ansell and Vonderheide 2013). The unique structure of the secondary lymphoid organs (including lymph nodes and spleen) in hematologic malignancies makes their microenvironment very different from that of solid tumors. In B-cell NHL, the TME is rich in immune cells, whereas in solid tumors, the number of infiltrating immune cells is relatively low (Ansell and Vonderheide 2013). Since the TME plays a crucial role in tumorigenesis, progression and recurrence, it is increasingly the focus of research on progression, metastasis and treatment resistance in solid and hematologic malignancies.

Here, we provide insight into the TME and tumor heterogeneity in DLBCL by analyzing single-cell transcriptomic data from 2 reactive lymph node tissue samples and 2 DLBCL lymph node biopsy tissue samples. We identified a high degree of inter-tumor heterogeneity in DLBCL samples and prominent immunosuppressive features in CD4^+^ regulatory T cells (CD4^+^ T_REG_) and exhausted cytotoxic CD8^+^ T cells (CD8^+^ T_EXH_). In addition, a prognostic model was constructed in a Bulk RNA-seq (Bulk-cell RNA sequencing) cohort containing 481 DLBCL samples based on the results of T cell subpopulation differential expression analysis, and the efficacy of the model in predicting prognosis and immunotherapy response was validated by the Gene Expression Omnibus (GEO) cohort and the Imvigor cohort.

## Materials and Methods

### Acquisition and Processing of scRNA-Seq Data

Single cell transcriptome data containing 2 reactive lymph node tissue samples and 2 DLBCL lymph node biopsy tissue samples were obtained from the heiDATA database (https://heidata.uni-heidelberg.de) (Supplementary Table S1). Single cell samples were prepared and Single-cell RNA sequencing (scRNA-seq) as follows: single cell suspensions, synthetic complementary DNA and single cell libraries were prepared using Chromium Single Cell v2 3ʹ kits (10x Genomics) according to the manufacturer’s instructions. Each was sequenced on a single NextSeq 550 lane (Illumina). The data were aligned to the hg38 reference genome with Cell Ranger (v2.1, 10x Genomics) using “mkfastq” and “count” commands and default parameters. The results of the Cell Ranger analysis contained the count values of unique molecular identifiers assigned to each gene in each of the cells for each individual sample using all mapped reads (Supplementary Table S2).

### Filtering of scRNA-Seq Data

The R package Seurat (v4.0.2) (Butler et al., 2018) was used to perform quality control. Gene counts per cell, UMI counts per cell, and percentages of mitochondrial and ribosomal transcripts were calculated using the functions of the Seurat package. Genes expressed in three or fewer cells were excluded from downstream analysis. Before further analysis, libraries with >5% of mitochondrial transcripts, libraries with UMI numbers indicating an abnormal range of potential doublets, and libraries with less than 200 genes were screened out. After removing low-quality cells, we analyzed scRNA-seq profiles of 11,729 cells with an average sequencing depth of approximately 1,400 genes per cell.

### Merging of Multisample Data With Correction for Batch Effects

The canonical correlation analysis (CCA) and mutual nearest neighbor (MNN) algorithms in the R package Seurat (v4.0.2) (Butler et al., 2018) were used for sample whole and correction of batch effects. After identifying the different cell types, the subsetdata function was used to split the dataset into subsets of different cell types.

### Clustering and Dimensionality Reduction

We used Seurat (v4.0.2) (Butler et al., 2018) to perform clustering analysis of cells. Data was normalized to log scale using the “NormalizeData” function with a default scale parameter of 10,000. “FindVariableFeatures” function was used to identify highly variable genes with parameters for “selection.method = vst, nfeatures = 2000”. We standardized the data with the “ScaleData” function. These variable genes were used as input for PCA using the “RunPCA” function. The first 20 principal components (PCs) and a resolution of 0.5 were used for clustering using “FindClusters”. Uniform manifold approximation and projection for dimension reduction (UMAP) was used for two-dimensional representation of first 20 PCs with “RunUMAP”. We used the “FindAllMarkers” or “FindMarkers” function to determine the marker genes of each cluster relative to all other clusters or to a specific cluster. The selected parameters of marker genes were detected in at least 25% of the cells in the target cluster, under *p* value of Wilcoxon test <0.05 and the differential expression threshold of 0.25 log fold change. FeaturePlot, DotPlot, VlnPlot and DoHeatmap were used for visualization of gene expression levels. We labeled the obtained clusters as T cells, B cells, NK cells, Dendritic cells (DC) and monocytes by known classical markers (T cells: CD3D, CD3E, CD3G, TRAC; B cells: MS4A1, CD79A; NK cells: NKG7, GNLY; DC: IRF7, IRF8; monocytes: LYZ, CD68.).

### Analysis of Intercellular Communication

Because DLBCL1 contains significantly more cells than DLBCL2, in order to perform a systematic analysis of intercellular communication, we re-clustered DLBCL1 for annotation and used the R package CellChat (v1.1.3) (Jin et al., 2021) to explore the expression of ligand-receptor pairs.

### Cell Trajectory Analysis

Branching developmental trajectories of CD8^+^ T cell subpopulations were calculated using the R package Monocle 2 (v2.16.0) (Qiu et al., 2017). Monocle introduces the strategy of ordering single cells in pseudo-time, by taking advantage of the asynchronous progression of individual cells in these processes and aligning them along trajectories corresponding to biological processes, such as cell differentiation.

### Single-Cell Regulatory Network Inference and Clustering Analysis

After annotation of each cell type by characterization of cell type marker genes, we used the SCENIC package (v1.2.4) (Aibar et al., 2017) to analyze the enriched transcription factors in cell subpopulations. The input matrix is a normalized expression matrix, output by Seurat.

### Gene Set Variation Analysis

Hallmark gene sets were downloaded from the MSigdb (Molecular Signatures Database) database and Gene Set Variation Analysis (GSVA) was performed using the R package GSVA to determine the molecular characteristics of different cell subpopulations. Gene-cell matrices are converted into gene set-cell matrices and GSVA scores are calculated for sets with at least 5 detected genes; all other parameters are default.

### Prognostic Model Construction and Validation

RNA-seq data and clinical information of 481 DLBCL patients were downloaded from The Cancer Genome Atlas (TCGA) database (https://cancergenome.nih.gov/) for screening prognostic genes and developing prognostic models. RNA sequencing data and clinical information for 420 DLBCL patients from the external validation cohort GSE10846 dataset were obtained from the GEO database. Data for the IMvigor210 immunotherapy cohort were obtained from the website http://research-pub.gene. com/IMvigor210CoreBiology. Extracted CD8^+^ T_EXH_ subpopulation-related genes obtained from differential gene expression analysis were used to construct prognostic models. In the TCGA cohort, univariate Cox regression analysis was performed using the R package Survival to screen prognosis-related genes (*p* < 0.05). Lasso regression analysis was performed using the R package glment to further screen prognosis-related genes, and finally six prognosis-related genes were obtained by multivariate Cox regression analysis for the construction of the prognostic risk model. The risk score of each patient was calculated as follows:

$$\mathrm{Risk score}=\sum_{j=1}^{n}\left(\mathrm{\beta j}\;\times \;\mathrm{expGj}\right)$$

where β is the regression coefficient obtained by multivariate Cox regression analysis and expG is the prognostic gene expression level. Based on the median risk scores obtained from the prognostic model, the DLBCL samples were divided into high-risk and low-risk groups, and survival differences between the different risk subgroups were compared by Kaplan-Meier curves. We plotted time-dependent subject operating characteristic (ROC) curves with 1, 3 and 5 years as the defined points, calculated the corresponding area under the ROC curve to assess the predictive power of the risk model, and verified whether the risk score was an independent prognostic indicator for DLBCL by Cox regression analysis. The GSE10846 cohort was used as an independent external validation cohort to verify the efficacy of the prognostic model.

### Tumor Microenvironment Score, Immune Cell Abundance and Immune Response Prediction

ESTIMATE is an algorithm that uses expression data to estimate stromal and immune cells in malignant tumor tissues, allowing estimation of stromal and immune scores for each DLBCL sample (Yoshihara et al., 2013). The deconvolution algorithm CIBERSORT is a method for characterizing cell composition from gene expression profiles of complex tissues, allowing inference of the relative content of immune cells from large amounts of tumor transcriptome data (Newman et al., 2015). Gene set enrichment analysis was performed using GSEA software (v4.1.0) to identify pathways that are predominantly enriched between high- and low-risk groups. Significantly enriched gene sets were screened with a threshold of *p* < 0.05. To validate the predictive power of prognostic models for immunotherapy response, the IMvigor210 immunotherapy cohort was used to assess differences in response to PD-L1 treatment in patients in different risk groups. Spearman correlation analysis was used to characterize the correlation between immune checkpoint genes and risk scores.

### Statistical Analysis

All statistical analyses were performed in R (v4.0.5). Comparisons between groups were performed using the Wilcoxon test and *t*-test. Correlations were analyzed by using Spearmans correlation. Survival curves were compared using log-rank test. Statistical significance was accepted for *p* < 0.05. *p < 0.05, **p < 0.01, ***p < 0.001.

## Results

### Single-Cell Transcriptomic Analysis Revealed the Complexity of Diffuse Large B-Cell Lymphoma

**FIGURE 1:**
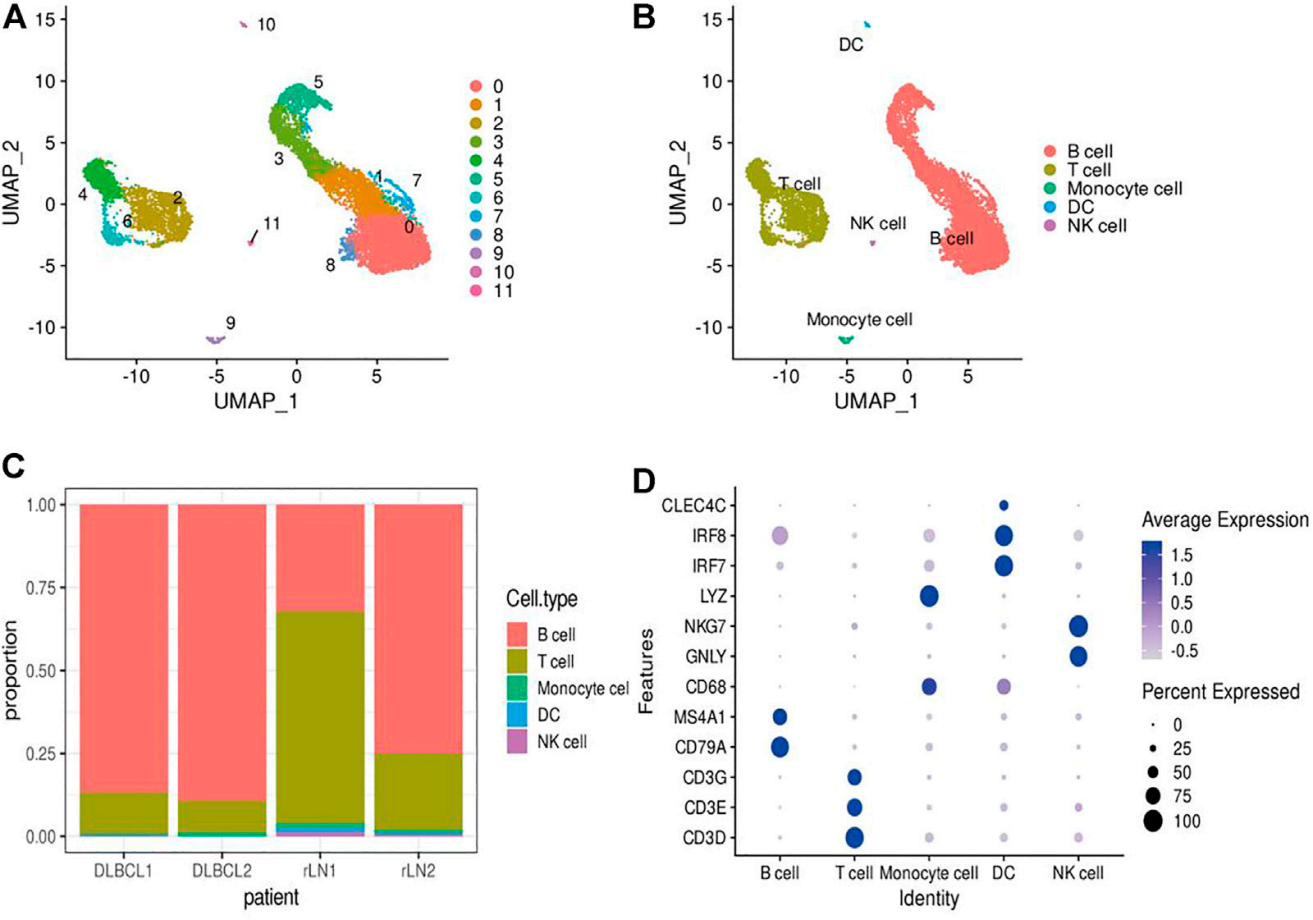
Identification of cell types using scRNA-seq. **(A,B)** Cells from 4 samples were combined and visualized using UMAP association. Cells were colored according to their cluster **(A)** or type **(B)**. **(C)** Bar graph showing the proportion of cell types in each sample. **(D)** Typical marker genes for the immune cell types defined in Figure 1B. Coloration was based on expression level.

In this study, single-cell transcriptomic data obtained from 10x Genomics sequencing were used to investigate the cellular diversity and molecular features in DLBCL tissues. After data quality control and filtering, 11,729 cells were obtained for subsequent analysis. After normalization of gene expression data, descending and clustering were performed using principal component analysis and UMAP, respectively. Twelve cell subpopulations were obtained by dimensionality reduction and clustering (Figure 1A), and these cells were assigned to five different cell types using known marker genes (Figures 1B,D): B cells (marker genes: MS4A1 and CD79A), T cells (marker genes: CD3D, CD3E, CD3G and TRAC), NK cells (marker genes: GNLY and NKG7), DC cells (marker genes: IR7 and IR8), monocytes (marker genes: LYZ and CD68). Notably, B cells and T cells are the major cell subsets of DLBCL (Figure 1C).

### Inter-Transcriptomic Heterogeneity of Malignant Cells in Diffuse Large B-Cell Lymphoma

**FIGURE 2:**
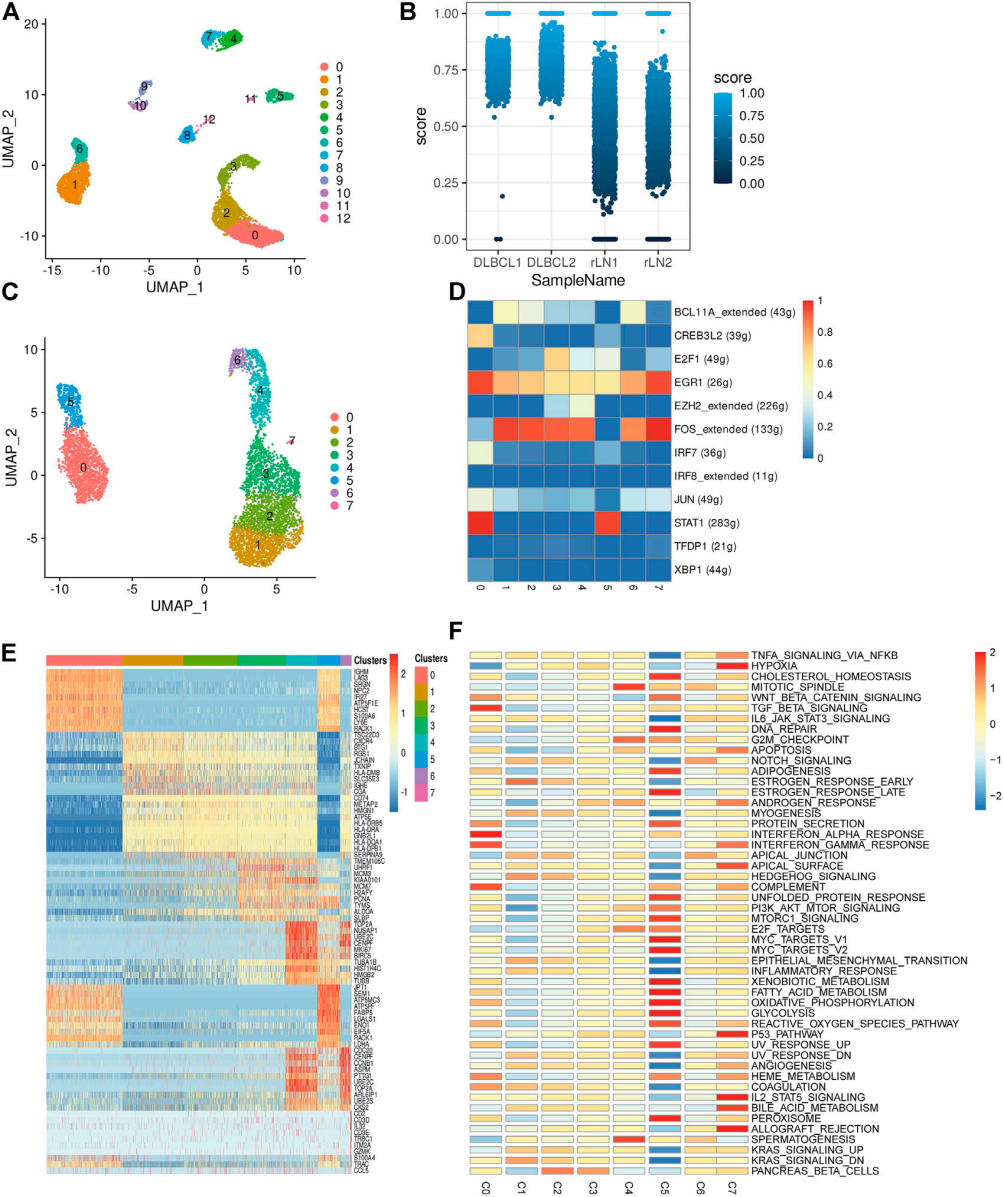
Transcriptome heterogeneity in malignant cells. **(A)** B cells from 4 samples were combined and visualized using UMAP association. Cells were colored according to their clusters. **(B)** IGKC fraction, IGKC ÷ (IGKC + IGLC2), was calculated for each B cell. B cells were classified as κ+ if the fraction was >0.5 and as λ+ if the ratio was below 0.5. The percentage of B cells expressing κ or λ was calculated based on the transcriptionally distinct B cell clusters. Nonmalignant B cells contain approximately 50% κ and 50% λ-expressing B cells, whereas malignant B cells contain B cells that uniformly express the κ light chain. **(C)** The umap plot of malignant B cells. **(D)** Heat map of area under the curve scores for regulation of expression by transcription factors imputed with SCENIC. **(E)** Heat map showing the top 10 differential genes in the 8 malignant B cell subpopulations (Wilcoxon test). **(F)** Differential activity pathways in the 8 malignant B cell subpopulations (scored by GSVA for each cell).

To investigate the transcriptomic heterogeneity of malignant B cells in DLBCL tissues, we re-clustered the B cells and identified 13 cell subpopulations. (Figure 2A). To further distinguish malignant B cells from non-malignant B cells, we took advantage of the fact that the malignant B cell population expresses only one type of immunoglobulin light chain, i.e. κ or λ light chains. The ratio of light chains per B cell (κ/λ) was calculated based on the expression of the genes IGKC (encoding a constant portion of the κ light chain) and IGLC2 (λ light chain). Malignant lymph nodes contain malignant B cells that uniformly express κ light chains, whereas reactive lymph node samples contain only non-malignant B cells (Figure 2B). We then re-clustered the malignant B cells and obtained eight malignant B cell subpopulations (Figure 2C), which showed a high degree of heterogeneity. SCENIC analysis identified EGR1, FOS and STAT1 as potential transcription factors (Figure 2D). Gene differential expression analysis revealed different transcriptional profiles among malignant B cell subpopulations: subpopulation 0 showed high expression levels of the malignancy-promoting factors S100A6 and LY6E, subpopulation 1 showed high expression levels of the tumor suppressor BTG1 and TXNIP, subpopulation 2 showed high expression levels of the immune-related genes CD74 and HLA-DRA, subpopulation 3 and subpopulation 4 showed high expression levels of cell proliferation genes MCM3, H2AFY, PCN, MKI67, TK1, subpopulation 5 showed high expression levels of metabolism-related genes FABP5, LDHA, ENO1, and subpopulation 6 showed high expression levels of cell cycle-related genes CENPF, CCNB1, CDC20 (Figure 2E). GSVA analysis showed different molecular signatures among malignant B-cell subpopulations: interferon response-dominant signature (subpopulation 0), cell proliferation-dominant signature (subpopulation 3 and subpopulation 4), metabolism-dominant signature (subpopulation 5), and hypoxia-dominant signature (subpopulation 7) (Figure 2F). In conclusion, these results reveal a high degree of inter-tumor heterogeneity in DLBCL.

### Enrichment of Immunosuppressive Tumor Infiltrating Regulatory T Cells in Diffuse Large B-Cell Lymphoma

**FIGURE 3:**
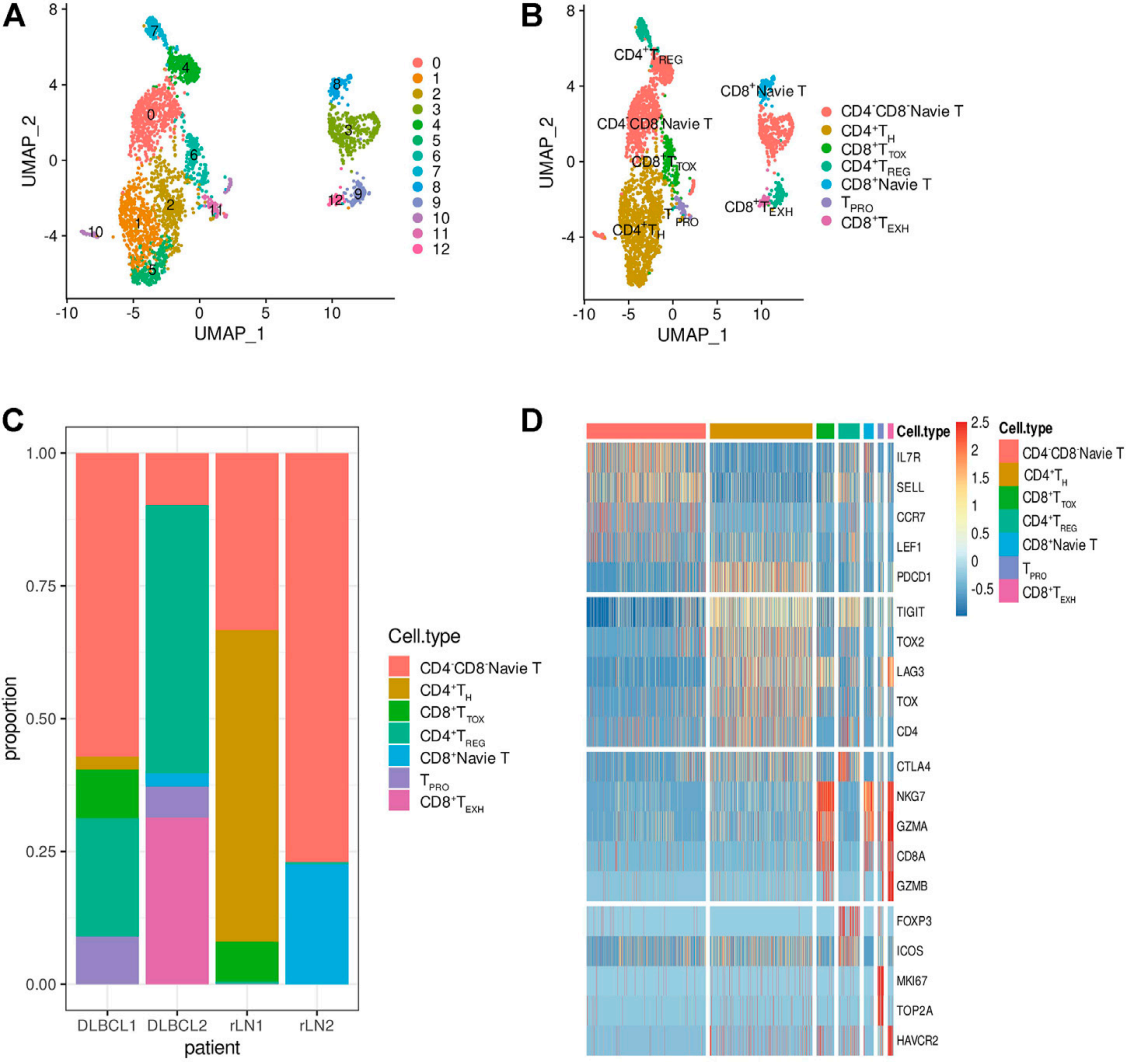
Transcriptome heterogeneity in T cells. **(A,B)** T cells from 4 samples were combined and visualized using UMAP association. Cells were colored according to their cluster or subtype. **(C)** Bar graph showing the proportion of cell types in each sample. **(D)** Differentially expressed genes used to identify T cell subpopulations.

Tumor-infiltrating immune cells are highly heterogeneous and play an important role in tumor cell immune evasion and response to immunotherapy. To investigate the transcriptomic heterogeneity of T cells in DLBCL tissues, we re-clustered T cells and identified 13 T cell subpopulations (Figures 3A–D). The T cell subpopulations were annotated by differentially expressed marker genes as: CD4^−^CD8^−^Navie T (IL7R,SELL, CCR7 and LEF1, subpopulations: 0, 3, 4 and 10), CD4^+^ T_H_ (CD4 and TRAC, subpopulations: 1, 2 and 5), CD8^+^ T_TOX_ (CD8A, GZMK and NKG7, subpopulation: 6), CD4^+^ T_REG_ (FOXP3,TIGIT, ICOS and CTLA4, subpopulations: 7 and 9), CD8^+^Navie T (CD8A, SELL and IL7R, subpopulation: 8), T_PRO_ (MKI67 and TOP2A, subpopulation: 11), CD8^+^ T_EXH_ (CD8A, GZMA, NKG7, LAG3 and HAVCR2, subpopulation: 12). To understand the state transitions between CD8^+^ T cell subtypes, we used Monocle2 to construct potential developmental trajectories of T cells. Developmental trajectories inferred from expression data or marker genes suggest (Supplementary Figures S1A, B) that CD8^+^ T cells have two differentiation pathways: cytotoxic CD8^+^ T cells (CD8^+^ T_TOX_) and exhausted CD8^+^ T cells (CD8^+^ T_EXH_). GSVA analysis revealed different signaling pathway enrichment among subpopulations: WNT and TGF signaling (CD4^+^ T_H_), TGF and TNF signaling (CD8^+^ T_TOX_), IL6/STAT3, IL2/STAT5 and KRAS signaling (CD4^+^ T_REG_), and interferon response (CD8^+^ T_EXH_) (Supplementary Figure S1C). SCENIC analysis identified SREBF2, RAD21, IRF7 as potential transcription factors in different T cell subpopulations (Supplementary Figure S1D). Taken together, our single-cell analyses reveal that CD4^+^ T_REG_ are highly immunosuppressive and CD8^+^ T_EXH_ highly express exhaustion markers such as LAG3, TIGIT and HAVCR2.

### Cellular Communication in Diffuse Large B-Cell Lymphoma

**FIGURE 4:**
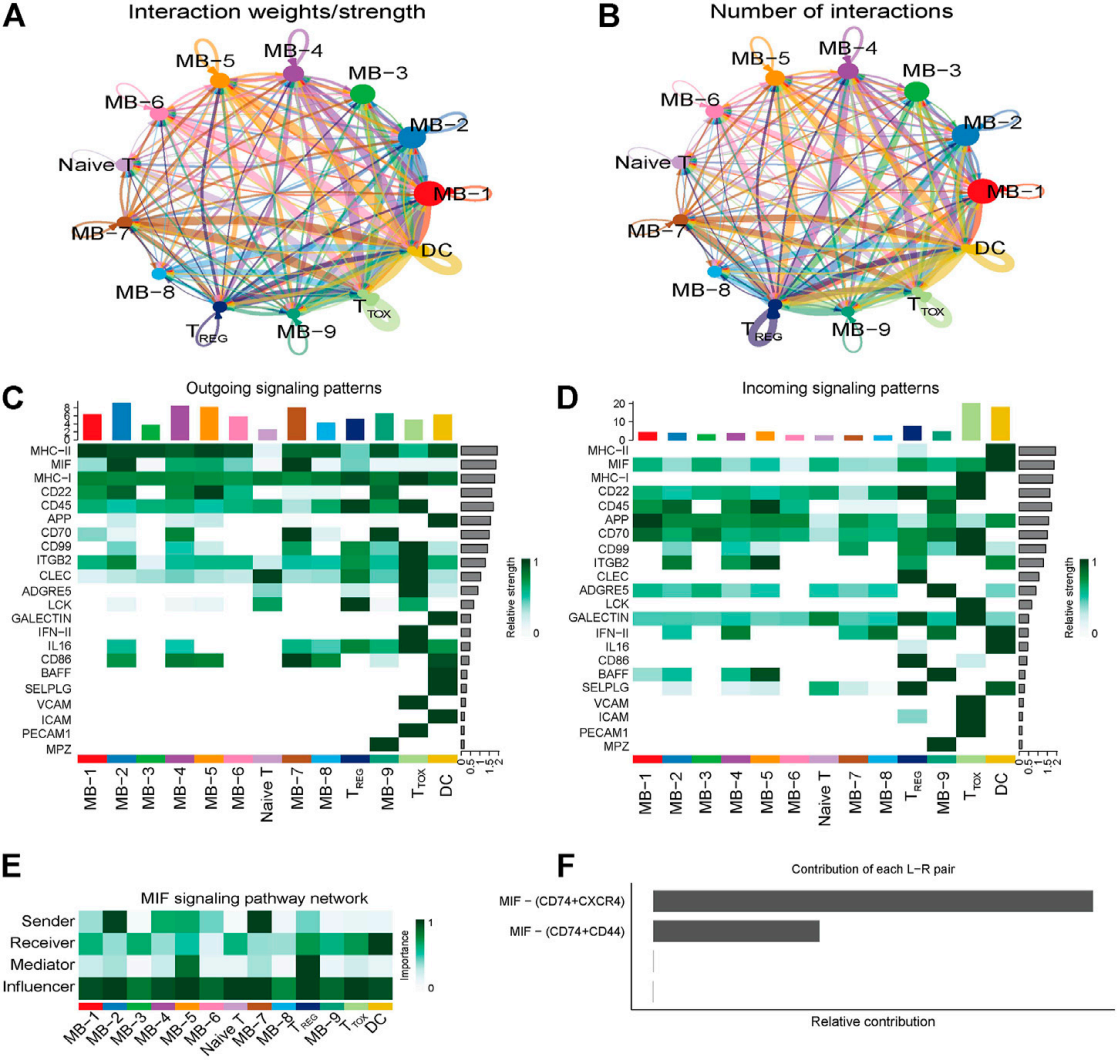
Cellular communication in DLBCL. **(A,B)** Circle diagram showing the number of interactions or strength of interactions between any two groups of cells. **(C,D)** Heat map of the cell-cell communication network for incoming or outgoing signaling action analysis. **(E)** Heat map showing the relative importance of each cell group based on the four network centrality degrees of the calculated MIF signaling network. **(F)** Relative contribution of each ligand-receptor pair to the overall communication network of the MIF signaling pathway.

**FIGURE 5:**
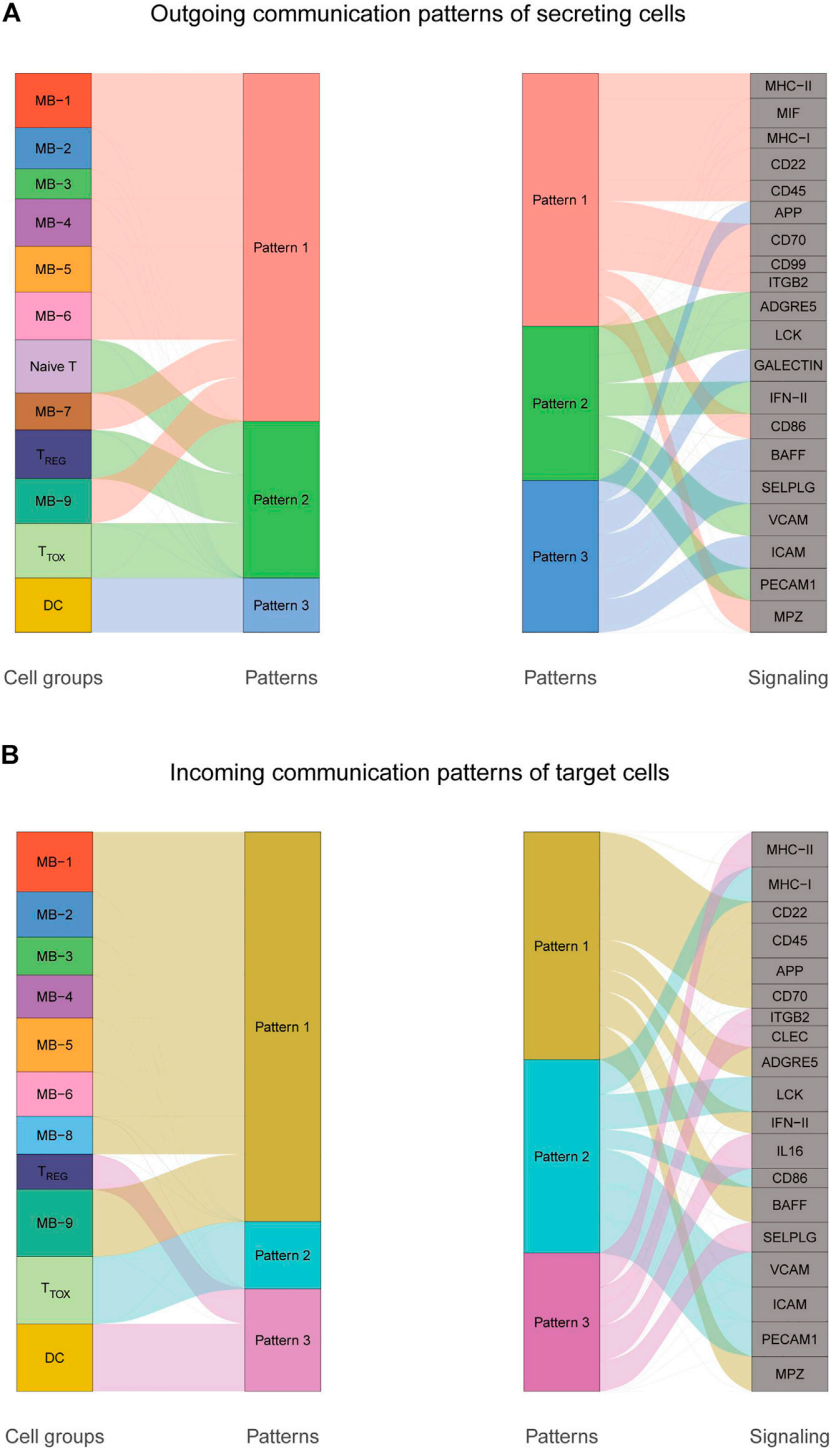
Cellular communication patterns in DLBCL. **(A)** Visualization of outgoing communication patterns of secretory cells by alluvial plots showing the correspondence between inferred potential patterns and cell populations, as well as signaling pathways. The thickness of the flow indicates the contribution of the cell population or signaling pathway to each potential pattern. The height of each pattern is proportional to the number of cell populations or signaling pathways associated with it. Outgoing communication patterns reveal how sending cells coordinate with each other and how they coordinate with certain signaling pathways to drive communication. **(B)** Incoming communication patterns of target cells. Incoming communication patterns reveal how target cells coordinate with each other and how they coordinate with certain signaling pathways in response to incoming signals.

To explore the interactions between cells in the DLBCL microenvironment, we used CellChat to infer and analyze intercellular communication networks. Dimension reduction, clustering and cell type annotation of sample DLBCL1 identified 13 cell subpopulations containing 9 malignant B cell subpopulations (MB1-9), 3 T cell subpopulations (T_REG_, T_TOX_, Naive T), and 1 DC cell subpopulation (DC). CellChat analysis revealed complex interactions between malignant B cell subpopulations and with other cell subpopulations, and 22 important pathways between 13 cell subpopulations were detected in DLBCL tissues, with the MIF signaling pathway being the prominent incoming and outgoing signaling mode (Figures 4A–D). Network centrality analysis of the inferred MIF signaling network showed that malignant B cell subpopulations (MB-2, MB-7) are the major senders and DCs are the major receivers of the MIF signaling pathway (Figure 4E). Notably, among all known ligand-receptor pairs, MIF signaling was predominantly dominated by the MIF ligand and its multimeric CD74/CXCR4 receptor (Figure 4F). CellChat uses a pattern recognition approach based on non-negative matrix decomposition to identify global communication patterns as well as key signals in different cell groups (i.e. pattern recognition modules). The output of this analysis is a set of the so-called communication patterns that connect cell groups with signaling pathways either in the context of outgoing signaling (i.e. treating cells as sources) or incoming signaling (i.e. treating cells as targets). The application of this pattern recognition module revealed three patterns of the outgoing signal and three patterns of the incoming signal (Figures 5A,B). The outgoing signaling of all malignant B cells is characterized by pattern #1, which includes the MHC-II, MIF, MHC-I, CD22, CD45 and other pathways, the outgoing signaling of T cells is characterized by pattern #2, which represents the ADGRE5, LCK, IFN-II, VCAM, PECAM1 and other pathways, and the outgoing signaling of DC is characterized by pattern #3, which includes the APP, BAFF, ICAM and other pathways. On the other hand, the communication patterns of target cells show that incoming malignant B cell signaling is dominated by patterns #1, which includes signaling pathways such as CD22, CD45, CD70, BAFF, IFN-II, etc. Incoming T cell signaling is characterized by two patterns #2 and #3, driven by pathways such as MHC-I, LCK, VCAM, ICAM, etc., while incoming DC signaling is also characterized by patterns #3. These results suggest that different cell types in the same tissue have different signaling networks and the pattern of malignant B-cell communication is homogeneous.

### Construction of a Prognostic Model Based on Exhausted CD8^+^ T Cell-Associated Genes

**FIGURE 6:**
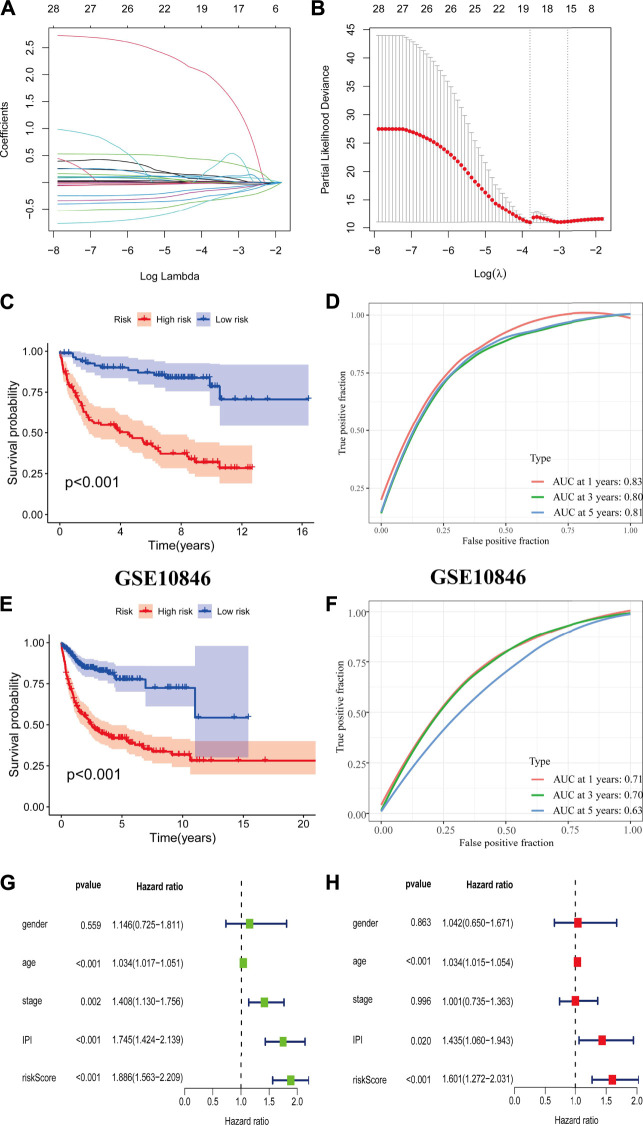
Construction and validation of prognostic model. **(A,B)** Coefficients of selected characteristics are shown by the lambda parameter, the horizontal axis represents the value of the independent variable lambda and the vertical axis represents the coefficient of the independent variable; partial likelihood deviation is plotted against log(λ) using the lasso Cox regression model. **(C,E)** Survival analysis curves for high and low risk score groups. **(D,F)** ROC curves of the prognostic model. **(G)** Univariate Cox regression analysis of DLBCL risk factors. **(H)** Multivariate Cox regression analysis of DLBCL risk factors.

The CD8^+^ T_EXH_ subpopulation-related genes obtained from the differential analysis were extracted for the construction of the prognostic model. Nineteen genes were obtained by univariate Cox regression analysis and lasso regression analysis (Figures 6A,B), and finally six prognosis-related genes for model construction were obtained using multivariate Cox regression analysis (GABRA3, HOXC8, RTN4R,CRLF1, BIRC3, REXO5). Using the regression coefficients for each of the above 6 prognostic genes, we constructed a prognostic model for DLBCL patients and calculated the risk score according to the following formula: risk score = (2.201 × GABRA3 expression level) + (−0.719 × HOXC8 expression level) + (−0.765 × RTN4R expression level) + (0.545 × CRLF1 expression level) + (−0.013 × BIRC3 expression level) + (−0.226 × REXO5 expression level). Using the median value of the risk score as the threshold, we divided DLBCL patients into low-risk and high-risk groups. Survival analysis showed that patients in the high-risk group had a poorer prognosis (*p* < 0.001) (Figure 6C), with an area under the ROC curve of 0.83, 0.80 and 0.80 for 1-year, 3-years and 5-years OS, respectively (Figure 6D). In the external validation cohort, survival analysis also showed a poorer prognosis for patients in the high-risk group (*p* < 0.001) (Figure 6E), with an area under the ROC curve of 0.71, 0.70 and 0.63 for 1-year, 3-years and 5-years OS, respectively (Figure 6F). The results of univariate and multivariate Cox regression analyses indicated that risk score was an independent prognostic factor (Figures 6G,H).

### Gene Set Enrichment Analysis for Different Risk Groups

**FIGURE 7:**
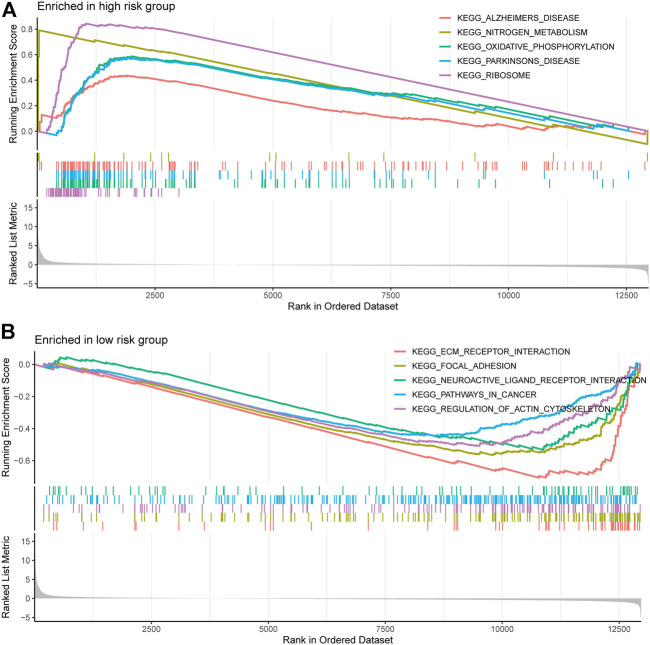
Gene and enrichment analysis of different risk groups. **(A)** KEGG-enriched pathway in the high-risk group (*p* < 0.05 and fdr-adjusted q < 0.05). **(B)** KEGG-enriched pathway in the low-risk group (*p* < 0.05 and fdr-adjusted q < 0.05).

We performed gene set enrichment analysis (GSEA) to identify potential biological processes between high- and low-risk groups. The results showed that pathways such as nitrogen metabolism, oxidative phosphorylation, ribosomes, Alzheimer’s disease, and Parkinson’s disease were enriched in the high-risk group, and pathways such as extracellular matrix receptor interactions, focal adhesion, gap linkage, pathways in cancer, and regulation of the actin cytoskeleton were enriched in the low-risk group (Figures 7A,B).

### Immune Landscape and Response to Immunotherapy in Different Risk Groups

**FIGURE 8:**
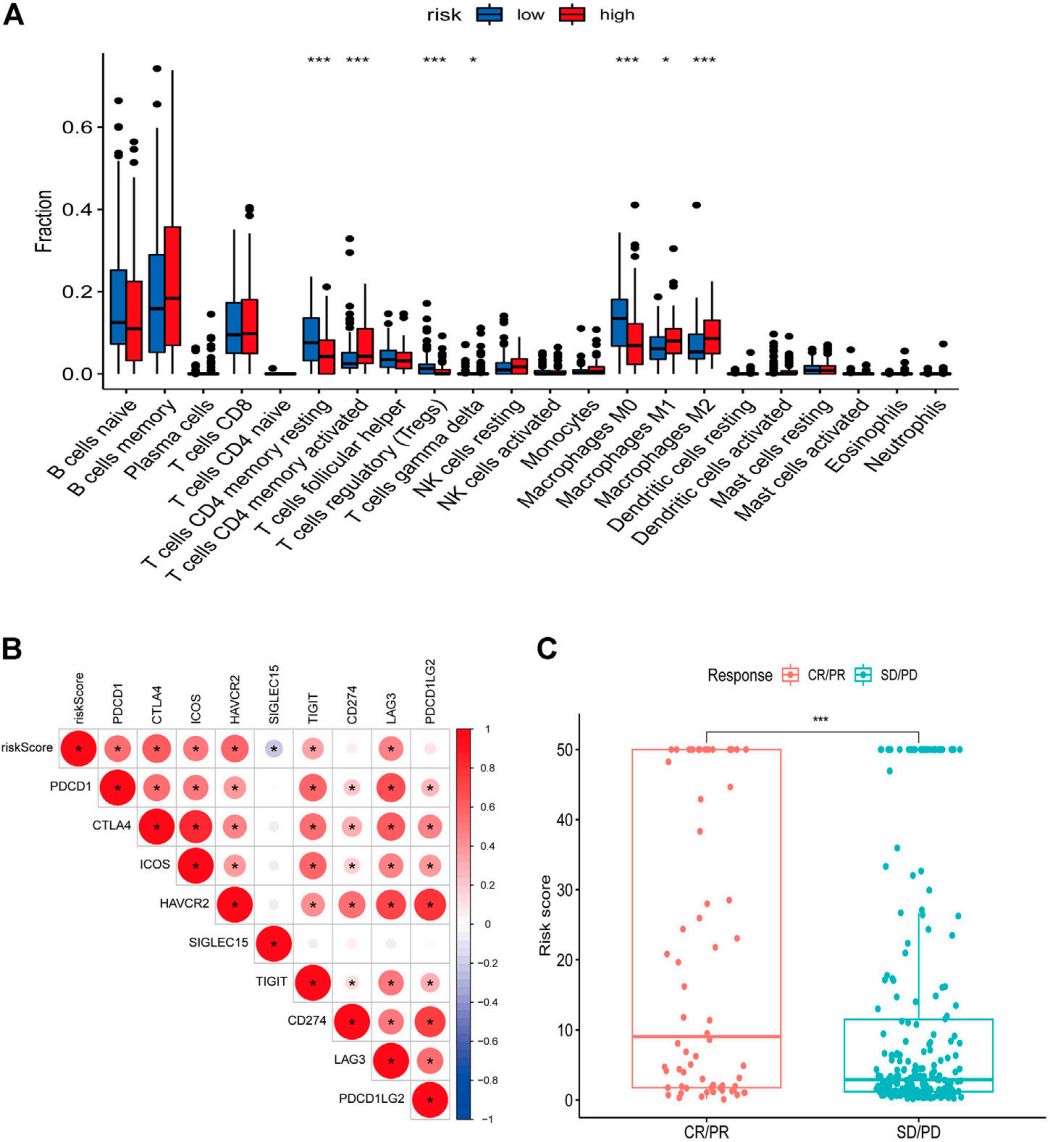
Relationship between risk score and immune landscape. **(A)** Distribution of 22 immune cell types in high and low risk groups. **(B)** Correlation matrix heat map showing the correlation analysis of risk scores with immune checkpoint genes. **(C)** Boxplot showing the difference in the distribution of risk scores in different immunotherapy response groups.

We used the ESTIMATE algorithm to assess the TME immune and stromal abundance in the different risk groups, and the results showed that the high-risk group had higher levels of immune and stromal component abundance (Supplementary Figures S2A–C). We also analyzed the proportion of 22 types of immune infiltrating cells among different risk groups in 481 DLBCL samples using the CIBERSORT algorithm (Supplementary Figure S2D), and the results showed that seven types of immune infiltrating cells were associated with risk scores: resting CD4 memory T cells, activated CD4 memory T cells, regulatory T cells, γδ T cells, and M0, M1, and M2 macrophages (Figure 8A). Correlation analysis of risk scores with immune checkpoint genes showed that most of the immune checkpoint gene expression levels were positively correlated with risk scores (Figure 8B). In addition, higher risk scores in the IMvigor210 immunotherapy cohort were associated with anti-PD-L1 treatment response (Figure 8C).

## Discussion

In this study, we combined scRNA-seq and bulk RNA-seq to investigate the tumor heterogeneity and TME characteristics of DLBCL. We showed the existence of malignant cell subpopulations with different transcriptional characteristics in DLBCL samples, such as a characteristic malignant cell subpopulation with predominantly cellular proliferation and a malignant cell subpopulation with predominantly metabolic characteristics. Roider T et al. investigated intra-tumor heterogeneity in B-NHL at the level of drug response by scRNA-seq, with tumor subgroups in the same lymph node responding significantly differently to targeted and chemotherapeutic agents (Roider et al., 2020). This suggests that a rational combination of anticancer drugs is needed to target all tumor subgroups, especially those with proliferative and aggressive characteristics, to improve therapeutic response and avoid the development of tumor drug resistance.

Immunotherapy has become a major hot topic in oncology treatment research, and inhibitors targeting the PD1-PDL1 axis have been approved as second- or first-line therapies for an increasing number of types of malignancies, including melanoma, lymphoma, lung cancer, renal cell carcinoma, head and neck squamous cell carcinoma, bladder cancer, liver cancer, and gastroesophageal cancer. However, great progress has been made in clinical application, but most patients receiving immune checkpoint inhibitors (ICIs) have not benefited from them (Gong et al., 2018). ICIs have shown significant efficacy in relapsed/refractory classic Hodgkin’s lymphoma (cHL), with an overall response rate (ORR) of 70–90% and have been approved for this indication (Ansell et al., 2015; Kasamon et al., 2017; Rossi et al., 2018). Unfortunately, ICIs are less effective in DLBCL, mainly due to its high biological heterogeneity. (Armand et al., 2013; Ansell et al., 2016; Lesokhin et al., 2016; Ansell et al., 2019; Frigault et al., 2020). By transcriptomic analysis of the microenvironment of multiple independent cohorts of DLBCL, Kotlov N et al. characterized four major lymphoma microenvironment (LME) categories associated with different biological abnormalities and clinical behaviors, namely GC-like, mesenchymal, inflammatory (IN), and depleted (DP) (Kotlov et al., 2021). Analysis of the correlation between LME category and response to chemoimmunotherapy showed that the number of responders was highest in GC-like patients and lowest in DP-LME patients. IN-LME is enriched in CD8^+^ T cells and a subpopulation of CD8^+^ T cells with high PD-1 expression and high expression of the immune checkpoint molecule PD-L1 and the tryptophanolytic enzyme IDO1, suggesting that this LME class may benefit from ICIs treatment. Steen CB et al. characterized clinically relevant DLBCL cell states and ecosystems with EcoTyper (a machine-learning framework integrating transcriptome deconvolution and single-cell RNA sequencing), identified 5 cell states of malignant B cells with different prognostic associations and differentiation status, and revealed nine multicellular ecosystems in DLBCL, known as lymphoma ecotypes (LE) (Steen et al., 2021). They found T-cell transcriptomic heterogeneity in DLBCL and that tumors high in LE4 are characterized by an immunoreactive T-cell state with widespread expression of co-inhibitory and stimulatory molecules, with potential implications for immunotherapeutic targeting. These studies suggest that exploring the heterogeneity of the DLBCL tumor microenvironment may better stratify patients to improve the efficacy of ICIs. Here, we identified seven different T cell subsets, CD4^−^CD8^−^Navie T, CD4^+^ T_H_, CD8^+^ T_TOX_, CD4^+^ T_REG_, CD8^+^Navie T, T_PRO_, and CD8^+^ T_EXH_. we found a significantly higher proportion of CD4^+^ T_REG_ cells in DLBCL samples compared to reactive lymph node tissue. Recently, several studies have found that CD4^+^FOXP3^+^ T cells can be divided into three subpopulations: 1) effector Tregs (eTregs), which have a strong suppressive function; 2) naive Tregs, which have the potential to differentiate into eTregs upon antigen stimulation; and 3) non-Tregs, which are a non-suppressive subpopulation (Nishikawa and Sakaguchi 2014). Studies have shown that high infiltration of FOXP3^+^ Tregs cells in DLBCL is associated with better prognosis, but these studies have targeted the entire FOXP3 population rather than the true Tregs cells (eTregs) that are essential for the impact of tumor immunity (Lee et al., 2008; Serag El-Dien et al., 2017). Nakayama S et al. found that high infiltration of FOXP3/CTLA-4 double-positive cells as eTregs was associated with a poorer prognosis (Nakayama et al., 2017). Recent animal studies with anti-CTLA-4 mAb using mice lacking antibody-dependent cytotoxic activity (by modulation of the Fc fraction or Fc receptor knockdown) showed that the anti-CTLA-4 mAb antitumor activity was attributed to depletion of FOXP3^+^CD4^+^ Treg cells from tumor tissue rather than direct activation of effector T cells (Bulliard et al., 2013; Selby et al., 2013; Simpson et al., 2013). Indeed, the reduction of FOXP3^+^CD4^+^ Treg cells in tumor tissue after anti-CTLA-4 mAb (Ipilimumab) treatment was strongly associated with clinical benefit (Hodi et al., 2008; Liakou et al., 2008). Furthermore, the critical role of CTLA-4 on FOXP3^+^CD4^+^ Treg cell function was revealed in animal studies, which showed that specific deletion of CTLA-4 in FOXP3^+^CD4^+^ Treg cells impairs their suppressive function and thus enhances antitumor immunity (Wing et al., 2008; Ise et al., 2010). Our single-cell analysis showed that the CD4^+^ T_REG_ subpopulation (highly expressing FOXP3 and CTLA-4) in DLBCL showed highly immunosuppressive properties, attributed to the eTregs, suggesting that immunotherapy against eTregs could be an effective and novel treatment strategy for DLBCL patients with highly infiltrated FOXP3/CTLA-4 double-positive cells.

In addition to the classical immune checkpoint molecules PD-1 and CTLA-4, T cell immunoglobulin mucin receptor 3 (TIM3, or HAVCR2) and LAG-3 are also included in the field of tumor immunotherapy research. TIM-3 is a type I transmembrane protein that is expressed on T cells in a number of malignancies, including melanoma, lung cancer, hepatocellular carcinoma, and colon cancer. In these tumors, TIM-3 expression is usually associated with dysfunctional T cells and poorer prognosis in some tumor types (Anderson 2014). In hematologic malignancies, TIM-3 expression has been observed in adult T-cell leukemia/lymphoma and extranodal NK/T-cell lymphoma (Horlad et al., 2016; Feng et al., 2018). In addition, TIM-3 expression levels in DLBCL patients have been found to correlate with tumor stage and response to chemotherapy (Xiao et al., 2014; Zhang et al., 2015). LAG-3 is a member of the immunoglobulin superfamily and functions as a negative regulator of T cell homeostasis. LAG-3 has been shown to be expressed in tumor-infiltrating lymphocytes in a variety of tumor types, including breast, ovarian, and lung cancers, and is commonly associated with increased numbers of PD-1^+^ T cells (Matsuzaki et al., 2010; Burugu et al., 2017; He et al., 2017). In follicular lymphoma, high expression of LAG-3 is associated with poorer patient prognosis and T-cell failure (Yang et al., 2017). Here, we characterized a population of CD8^+^ T cells with high expression of LAG-3, TIM-3, TIGHT, i.e. exhausted cytotoxic CD8^+^ T cells, which showed a molecular profile dominated by interferon response and retained the expression of GZMA, GZMB and NKG7. Furthermore, by SCENIC analysis, we revealed potential transcription factors, such as STAT1 and IRF7, in the CD8^+^ T_EXH_ cell subpopulation. Beltra JC et al. showed that in exhausted CD8^+^ T cells are enriched with open chromatin regions that bind to STAT1 and IRF7 (Beltra et al., 2020), which is consistent with our findings.

We constructed prognostic models based on differential genes associated with CD8^+^ T_EXH_ subpopulations obtained from previous differential gene expression analysis, and the efficacy of the prognostic models in predicting survival, and response to immunotherapy was validated by internal or external validation cohorts. This prognostic model could identify high-risk DLBCL patients and helped clinicians make better clinical decisions.

In conclusion, this study provides an in-depth dissection of the transcriptional features of malignant B cells and TME in DLBCL and provides new insights into the tumor heterogeneity of DLBCL. The data from our study can serve as a resource for subsequent in-depth studies to provide therapeutic targets and biomarkers for immunotherapy in DLBCL through deeper biological exploration. In addition, the prognostic model we developed can well predict the prognostic status and immunotherapeutic response of DLBCL patients with promising clinical applications.

## Data Availability

The original contributions presented in the study are included in the article/Supplementary Material, further inquiries can be directed to the corresponding author.
