# Supplementary material for: Single-Cell RNA-Seq and Bulk RNA-Seq Reveal Intratumoral Heterogeneity and Tumor Microenvironment Characteristics in Diffuse Large B-Cell Lymphoma
Source: Front Genet. 2022 May 4;13:881345. doi: 10.3389/fgene.2022.881345 (PMC9116505; doi:10.3389/fgene.2022.881345)
Supplement: Supplementary file 2 [file DataSheet1.docx]

Supplementary Material

## Supplementary Figures


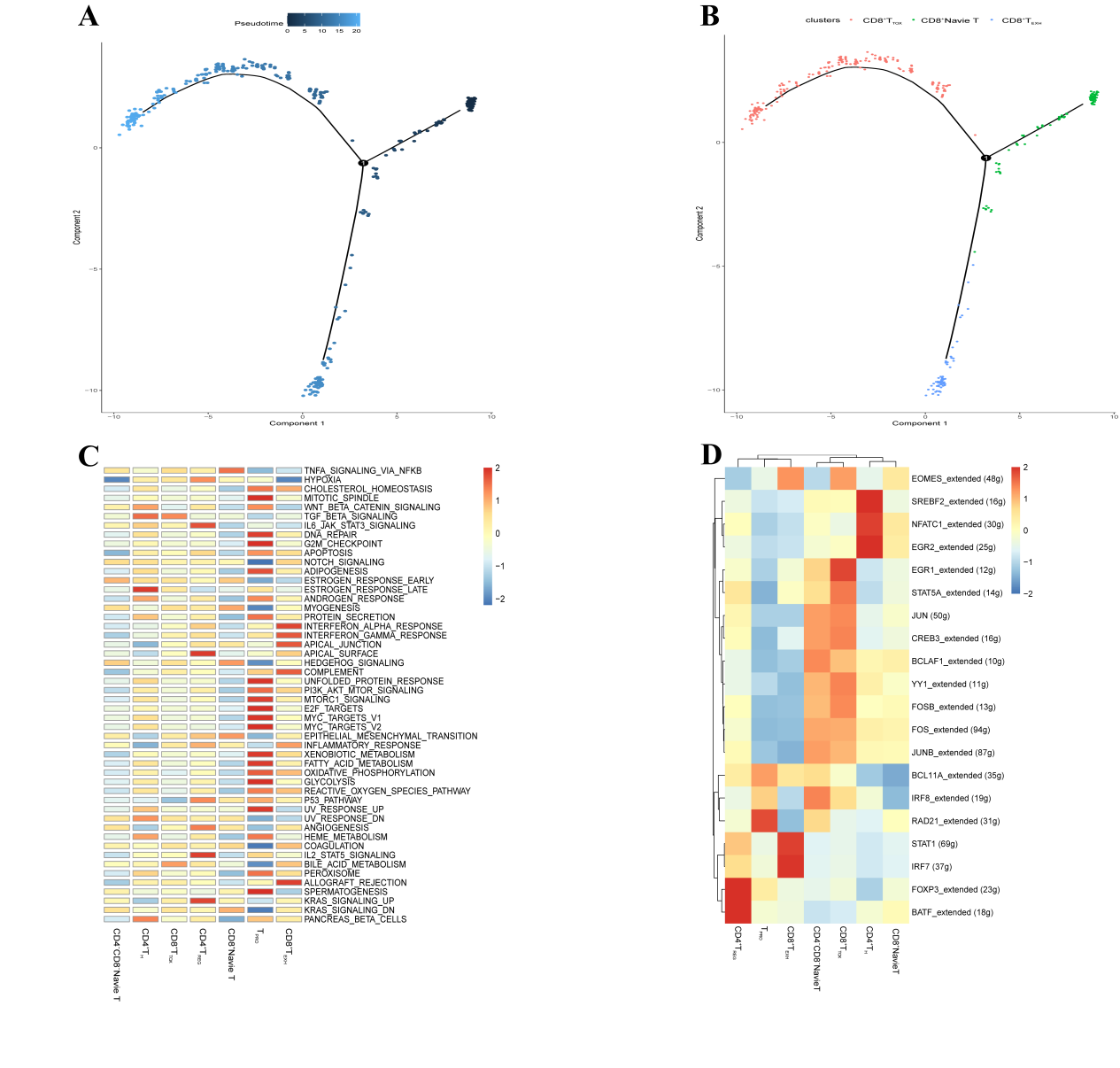


**Supplementary Figure 1.** Transcriptome heterogeneity in T cells. (A, B) Trajectory diagram of the proposed chronological analysis of CD8^+^ T cells. (C) Heat map showing differential activity pathways in 7 T cell subpopulations (scored by GSVA for each cell). (D) Heat map showing area under the curve scores of SCENIC-derived transcription factors on expression regulation.


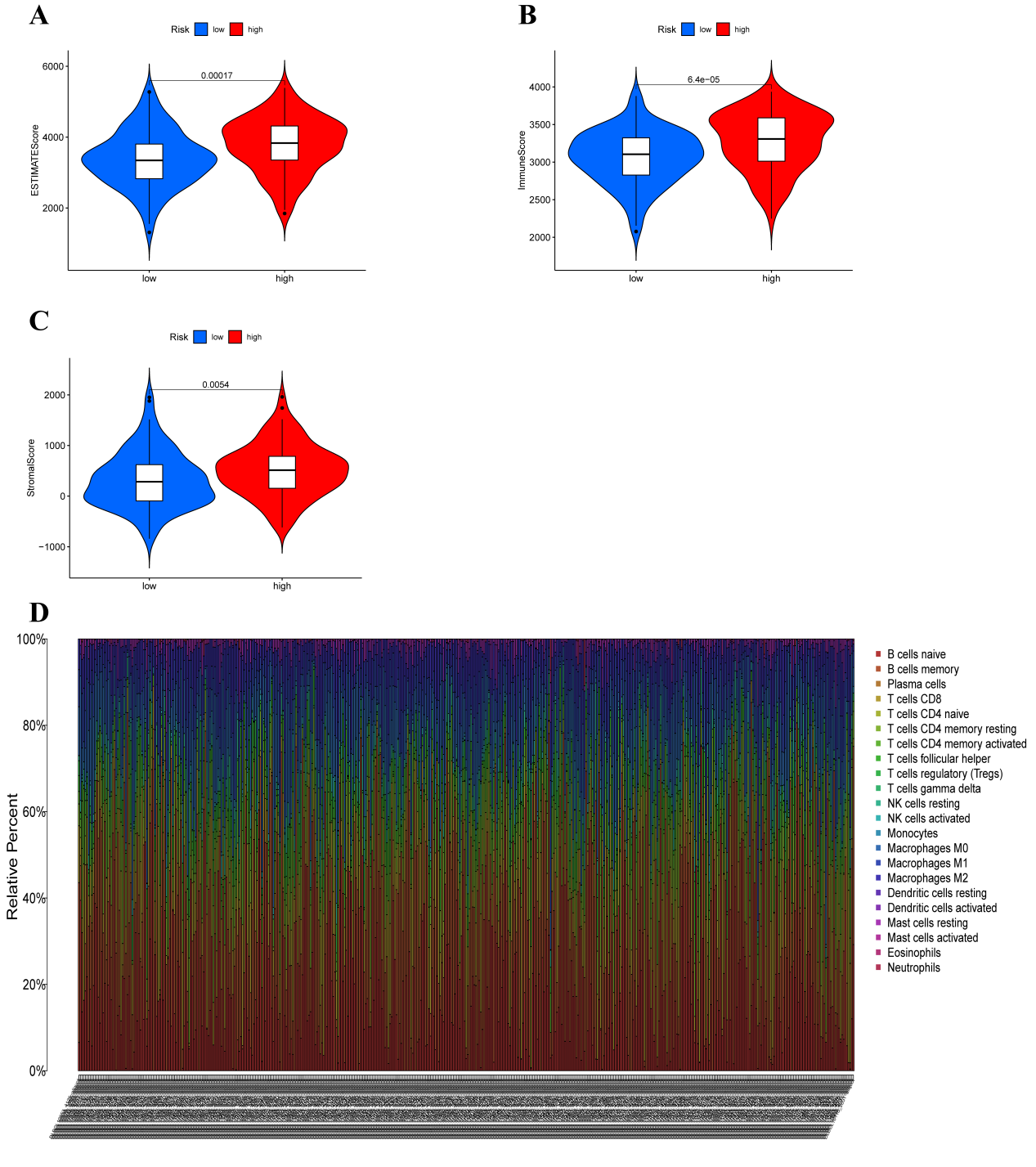


**Supplementary Figure 2.** Immune landscape of DLBCL. (A, B, C) Violin plots showing the differences in the distribution of immune score, stromal score, and estimated score in high and low risk populations. (D) Bar graph showing the proportion of 22 TICs in DLBCL tumor samples.
